# Characterization of the neurohypophysial hormone gene loci in elephant shark and the Japanese lamprey: origin of the vertebrate neurohypophysial hormone genes

**DOI:** 10.1186/1471-2148-9-47

**Published:** 2009-02-26

**Authors:** Pai-Chung Gwee, Boon-Hui Tay, Sydney Brenner, Byrappa Venkatesh

**Affiliations:** 1 Institute of Molecular and Cell Biology, A*STAR (Agency for Science, Technology and Research), Biopolis, 138673 Singapore

## Abstract

**Background:**

Vasopressin and oxytocin are mammalian neurohypophysial hormones with distinct functions. Vasopressin is involved mainly in osmoregulation and oxytocin is involved primarily in parturition and lactation. Jawed vertebrates contain at least one homolog each of vasopressin and oxytocin, whereas only a vasopressin-family hormone, vasotocin, has been identified in jawless vertebrates. The genes encoding vasopressin and oxytocin are closely linked tail-to-tail in eutherian mammals whereas their homologs in chicken, *Xenopus *and coelacanth (*vasotocin *and *mesotocin*) are linked tail-to-head. In contrast, their pufferfish homologs, *vasotocin *and *isotocin*, are located on the same strand of DNA with *isotocin *located upstream of *vasotocin *and separated by five genes. These differences in the arrangement of the two genes in different bony vertebrate lineages raise questions about their origin and ancestral arrangement. To trace the origin of these genes, we have sequenced BAC clones from the neurohypophysial gene loci in a cartilaginous fish, the elephant shark (*Callorhinchus milii*), and in a jawless vertebrate, the Japanese lamprey (*Lethenteron japonicum*). We have also analyzed the neurohypophysial hormone gene locus in an invertebrate chordate, the amphioxus (*Branchiostoma floridae*).

**Results:**

The elephant shark neurohypophysial hormone genes encode vasotocin and oxytocin, and are linked tail-to-head like their homologs in coelacanth and non-eutherian tetrapods. Besides the hypothalamus, the two genes are also expressed in the ovary. In addition, the *vasotocin *gene is expressed in the kidney, rectal gland and intestine. These expression profiles indicate a paracrine role for the two hormones. The lamprey locus contains a single neurohypophysial hormone gene, the *vasotocin*. The synteny of genes in the lamprey locus is conserved in elephant shark, coelacanth and tetrapods but disrupted in teleost fishes. The amphioxus locus encodes a single neurohypophysial hormone, designated as [Ile^4^]vasotocin.

**Conclusion:**

The vasopressin- and oxytocin-family of neurohypophysial hormones evolved in a common ancestor of jawed vertebrates through tandem duplication of the ancestral *vasotocin *gene. The duplicated genes were linked tail-to-head like their homologs in elephant shark, coelacanth and non-eutherian tetrapods. In contrast to the conserved linkage of the neurohypophysial genes in these vertebrates, the neurohypophysial hormone gene locus has experienced extensive rearrangements in the teleost lineage.

## Background

Neurohypophysial hormones are an ancient family of structurally and functionally related nonapeptides, with representatives found in deuterostomes as well as in protostomes. Vasopressin and oxytocin are mammalian neurohypophysial hormones with distinct activities: vasopressin has renal urine reabsorption (antidiuretic) and blood-pressure raising (vasopressor) activities while oxytocin has uterus-contracting (uterotonic) and milk-ejecting (galactagogic) activities. Vasopressin is a basic peptide while oxytocin is a neutral peptide, a characteristic determined mainly by the amino acid at the 8^th ^position. All jawed vertebrates contain at least one vasopressin-family, basic peptide and one oxytocin-family, neutral peptide whereas only a vasopressin-family peptide has so far been identified in jawless vertebrates such as lamprey and hagfishes [1,2]. Vasotocin is the vasopressin-family peptide in all non-mammalian vertebrates. Oxytocin-family peptides, however, exhibit a wide diversity. The oxytocin homolog in non-eutherian tetrapods and lobe-finned fishes (lungfish and coelacanth) is mesotocin and in ray-finned fishes is isotocin. The cartilaginous fishes (sharks, rays, skates and chimaeras) contain at least eight types of oxytocin-family peptides. They are asvatocin and phasvatocin in the spotted dogfish (*Scyliorhinus canicula*); asvatocin and phasitocin in the Japanese banded dogfish (*Triakis scyllium*); valitocin and aspargtocin in the spiny dogfish (*Squalus acanthias*); glumitocin in skates (*Raja*); isotocin in the electric ray (*Torpedo mormorata*); and oxytocin in a holocephalian chimaera, the ratfish (*Hydrolagus colliei*) (see Table 1). It should be noted that these neuropeptides are named according to certain unique residues and their biochemical properties and not according to any phylogenetic grouping. For example, oxytocin molecule is present in placental mammals and in a cartilaginous fish, the ratfish. The presence of oxytocin in these distantly related vertebrates is the result of independent nucleotide substitutions in the two lineages.

**Table 1:** Neurohypophysial hormones in vertebrates

*Vasopressin homologs*		
Vasopressin	Cys-Tyr-Phe-Gln-Asn-Cys-Pro-Arg-Gly (NH_2_)	Mammals
[Lys^8^]vasopressin	Cys-Tyr-Phe-Gln-Asn-Cys-Pro-Lys-Gly (NH_2_)	Pigs and some marsupials
[Phe^2^]vasopressin	Cys-Phe-Phe-Gln-Asn-Cys-Pro-Arg-Gly (NH_2_)	Some marsupials
Vasotocin	Cys-Tyr-Ile-Gln-Asn-Cys-Pro-Arg-Gly (NH_2_)	Non-mammals

*Oxytocin homologs*		

Oxytocin	Cys-Tyr-Ile-Gln-Asn-Cys-Pro-Leu-Gly (NH_2_)	Placental mammals, some marsupials, platypus, ratfish (*H. colliei*), elephant shark
Mesotocin	Cys-Tyr-Ile-Gln-Asn-Cys-Pro-Ile-Gly (NH_2_)	Some marsupials, non-mammalian tetrapods, some lungfishes, coelacanth
[Phe^2^]mesotocin	Cys-Phe-Ile-Gln-Asn-Cys-Pro-Ile-Gly (NH_2_)	Australian lungfish
Isotocin	Cys-Tyr-Ile-Ser-Asn-Cys-Pro-Ile-Gly (NH_2_)	Ray-finned fishes, marbled electric ray (*T. marmorata*)
Glumitocin	Cys-Tyr-Ile-Ser-Asn-Cys-Pro-Gln-Gly (NH_2_)	Skates
Valitocin	Cys-Tyr-Ile-Gln-Asn-Cys-Pro-Val-Gly (NH_2_)	Sharks (*Sq. acanthias*)
Aspargtocin	Cys-Tyr-Ile-Asn-Asn-Cys-Pro-Leu-Gly (NH_2_)	Sharks (*Sq. acanthias*)
Asvatocin	Cys-Tyr-Ile-Asn-Asn-Cys-Pro-Val-Gly (NH_2_)	Sharks (*Sc. canicula; T. scyllium*)
Phasitocin	Cys-Tyr-Phe-Asn-Asn-Cys-Pro-Ile-Gly (NH_2_)	Sharks (*T. scyllium*)
Phasvatocin	Cys-Tyr-Phe-Asn-Asn-Cys-Pro-Val-Gly (NH_2_)	Sharks (*Sc. canicula*)

Adapted from [18,37,38].

**Figure 1:**
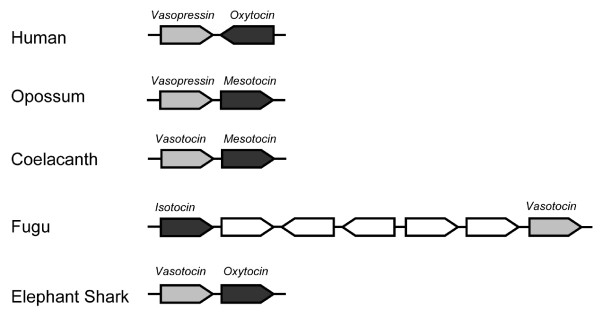
**Organization of neurohypophysial hormone-genes in jawed vertebrates**. Genes are shown as arrows. Only the neurohypophysial hormone-genes are labeled. The elephant shark genes were sequenced in this study. The arrangements of genes in other vertebrates are mainly from [8]. The neurohypophysial hormone-genes in chicken and *Xenopus *are arranged like their homologs in coelacanth.

The neurohypophysial hormones are synthesized as part of a larger precursor molecule comprising a signal peptide, the nonapeptide hormone, and a neurophysin. The precursors of the vasopressin-family hormones and the isotocin hormone in teleost fishes contain an additional peptide, the copeptin, at the carboxyl terminal. In placental mammals, the genes encoding vasopressin and oxytocin are closely linked in a tail-to-tail orientation. Nevertheless, the two genes are expressed in distinct magnocellular neurons of the supraoptic nuclei and paraventricular nuclei of the hypothalamus. In addition, vasopressin is expressed in the parvocellular neurons of the paraventricular nuclei and suprachiasmatic nuclei [3]. The *cis*-regulatory elements that mediate the hypothalamus-specific expression of the two genes have been shown to be located in their intergenic region [4-7]. In contrast to the tail-to-tail arrangement of vasopressin and oxytocin genes in placental mammals, their homologs in opossum, chicken, *Xenopus *and coelacanth are located on the same strand of DNA and are closely linked in a tail-to-head orientation (Fig 1). Interestingly, although their homologs in pufferfishes (*vasotocin *and *isotocin*) reside on the same strand of DNA, they are arranged in a different order: *isotocin *gene is located upstream of *vasotocin *gene and separated by five unrelated genes (Fig 1) [8]. These marked differences in the organization of the vasopressin- and oxytocin-family genes in different bony vertebrate lineages raise questions about their origin and the organization of the ancestral vasopressin-family and oxytocin-family genes.

The cartilaginous fishes are the oldest group of living jawed vertebrates that diverged from bony vertebrates about 450 million years ago [9]. The ancestor of jawed vertebrates split from jawless vertebrates about 477 million years ago [10]. The neurohypophysial hormone gene locus has not been sequenced from either cartilaginous fishes or jawless fishes. Characterization of the neurohypophysial hormone gene loci in these vertebrates should shed light on the origin and organization of the ancestral vasopressin- and oxytocin-family genes. In this study, we have sequenced and characterized the neurohypophysial hormone gene loci in a cartilaginous fish, the elephant shark (*Callorhinchus milii*) and in a jawless vertebrate, the Japanese lamprey (*Lethenteron japonicum*). We have also identified and analyzed the neurohypophysial hormone gene in the recently completed genome sequence of the amphioxus (*Branchiostoma floridae*), an invertebrate chordate which represents the most basal group of living chordates (the cephalochordates).

The cartilaginous fishes are divided into two groups: elasmobranchs (sharks, rays and skates) and holocephalians (chimaeras). The elephant shark is a holocephalian chimaera that inhabits the continental shelves off southern Australia and New Zealand at depths of 200 to 500 meters. We chose elephant shark as a representative cartilaginous fish because it has the smallest genome (910 Mb) among cartilaginous fishes [11,12]. Like lungfishes and coelacanths, cartilaginous fishes conduct urea-based osmoregulation. Cartilaginous fishes, particularly the marine species, maintain their plasma iso-osmotic or slightly hyper-osmotic to the seawater mainly through the retention of urea. In addition to the gut, kidney and gills, which are the major osmoregulatory organs in fishes, marine cartilaginous fishes contain a fourth osmoregulatory organ, the rectal gland that is devoted exclusively to sodium chloride excretion. Interestingly, elephant shark does not contain a discrete rectal gland like the marine elasmobranchs. Instead, its rectal gland comprises about 10 tubular structures located in the wall of post-valvular intestine [13]. However, elephant shark maintains its plasma levels of Na (~300 mmol l^-1^) and urea (~450 mmol l^-1^) similar to those in marine elasmobranchs [13].

## Results and discussion

### Neurohypophysial hormone gene locus in the elephant shark

**Figure 2:**
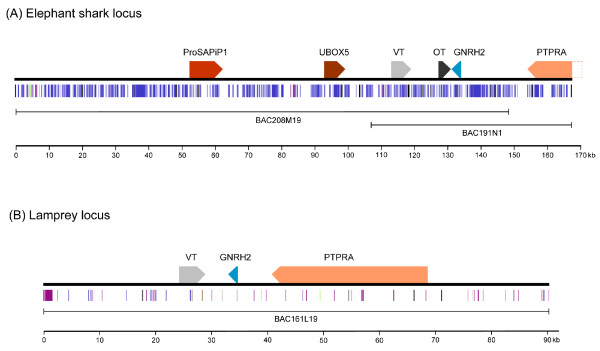
**The neurohypophysial gene locus in the elephant shark (A) and Japanese lamprey (B)**. The BACs used for generating the sequences are shown below. Arrows represent genes and indicate the direction of transcription. Coloured vertical lines represent repetitive sequences. VT, *vasotocin *gene; OT, *oxytocin *gene; ProSAPiP1, *proline rich synapse associated protein interacting protein 1*; UBOX5, *U-box domain containing 5*; GNRH2, *gonadotropin-releasing hormone 2*; PTPRA, *protein tyrosine phosphatase, receptor type, A*.

We probed an elephant shark BAC library with a fragment of the elephant shark vasotocin gene and identified six overlapping BAC clones. Two of the BAC clones, #191N1 and #208M19, were sequenced completely to obtain 167 kb contiguous sequence (GenBank accession number FJ185172). It contains sequences for *vasotocin *and *oxytocin *genes in addition to three other complete genes (*Prosapip1*, *Ubox5 *and *Gnrh2*) and one partial gene (*Ptpra*) (Fig 2A). Oxytocin is typically only found in placental mammals and is involved in lactation, uterine smooth muscle contraction and maternal behavior. However, oxytocin had been previously purified from the hypothalamus of a holocephalian cartilaginous fish, the ratfish, the only non-mammalian vertebrate to contain oxytocin [14]. The presence of *oxytocin *gene in elephant shark indicates that oxytocin is most likely common to all holocephalian cartilaginous fishes. The elephant shark *vasotocin *and *oxytocin *genes are arranged tail-to-head like their homologs in coelacanth, *Xenopus*, chicken and opossum. They each comprise three exons and two introns like their homologs in other vertebrates. The introns of elephant shark *vasotocin *and *oxytocin *genes (1.16 kb to 3.24 kb) are longer than their homologs in human (84 bp to 1.37 kb) but comparable to that in coelacanth (1.55 kb to 5.57 kb). The intergenic distance between the elephant shark genes is, however, shorter (8.3 kb) than that between the genes in human (12 kb) and coelacanth (15.4 kb). Overall, repetitive sequences account for 41.6% of the elephant shark locus with LINEs and SINEs contributing 16.9% and 20.1% respectively (Fig 2A).

### Elephant shark vasotocin and oxytocin precursors

**Figure 3:**
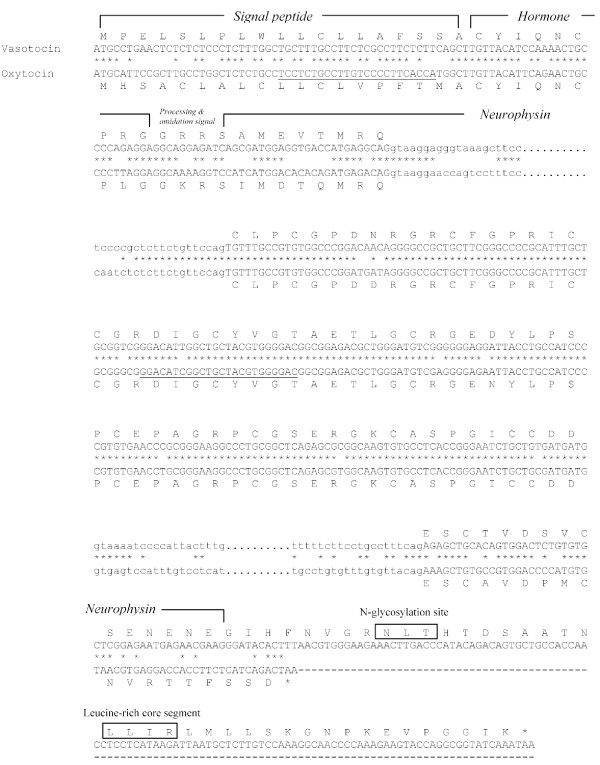
**Comparison of nucleotide and deduced amino acid sequences of elephant shark vasotocin and oxytocin genes**. Conserved nucleotides are indicated by an asterisk. N-glycosylation sites and Leu-rich core segment in the vasotocin precursor are boxed. Intronic sequences are shown in lower case.

**Figure 4:**
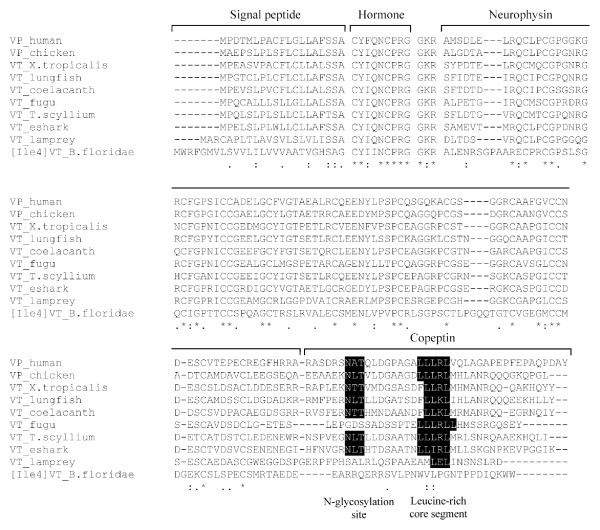
**Comparison of amino acid sequences of vasopressin family hormone precursors in vertebrates**. The alignment was generated by ClustalX. Amino acid residues conserved in all vertebrates are marked with an asterisk. *B. floridae, Branchiostoma floridae; X. tropicalis*, *Xenopus tropicalis*; and *T. scyllium*, *Triakis scyllium*. VP, vasopressin and VT, vasotocin. Accession numbers of sequences used in the alignment: NP_000481.2 (human VP), BAA24026.1 (lungfish VT), O42499 (fugu VT), BAD27476.1 (*T. scyllium *VT) and BAA06669.1 (lamprey VT). Sequences for *Xenopus tropicalis *and coelacanth were generated in a previous study [8] while sequences for elephant shark (eshark) and amphioxus (*B. floridae*) were generated in this study.

The elephant shark *vasotocin *gene codes for a 163-amino acid protein comprising a signal peptide, the vasotocin nonapeptide, a neurophysin and a copeptin similar to vasotocin precursors in other vertebrates (Fig 3). An atypical tripeptide sequence, Gly-Arg-Arg, links the hormone to the neurophysin and presumably acts as a signal for proteolytic processing and carboxyl-terminal amidation of vasotocin. All the cysteine residues that are considered important for the conformation of neurophysin are conserved in the elephant shark vasotocin neurophysin (Fig 4). The copeptin moiety at the carboxyl terminal includes an N-linked glycosylation site that is conserved in all vertebrates except teleost fishes and lamprey (Fig. 4). It also includes a leucine-rich core segment similar to the copeptin of vasopressin-family precursors in all vertebrates (Fig 4).

**Figure 5:**
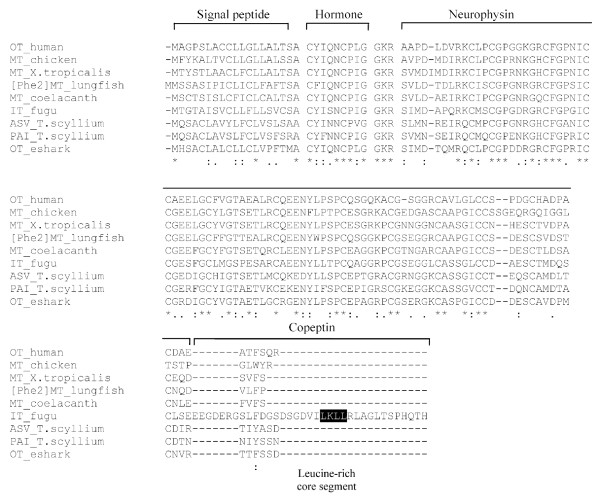
**Comparison of amino acid sequences of oxytocin family hormone precursors in vertebrates**. The alignment was generated by ClustalX. Amino acid residues conserved in all vertebrates are marked with an asterisk. *X. tropicalis*, *Xenopus tropicalis*; and *T. scyllium*, *Triakis scyllium*. OT, oxytocin; MT, mesotocin; IT, isotocin; ASV, asvatocin and PAI, phasitocin. Accession numbers of sequences used in the alignment: NP_000906.1 (human OT), BAA24027.1 (lungfish [Phe^2^]MT), O42493 (fugu IT), BAD27478.1 (*T. scyllium *phasitocin) and BAD27477.1 (*T. scyllium *asvatocin). Sequences for *Xenopus tropicalis *and coelacanth were generated in a previous study [8] while the sequence for elephant shark (eshark) was generated in this study.

The elephant shark *oxytocin *gene codes for a shorter 126-amino acid protein which includes a signal peptide, the oxytocin nonapeptide and a neurophysin (Fig 3). Oxytocin is attached to the neurophysin via a typical tripeptide sequence Gly-Lys-Arg that is known to act as a signal for proteolytic processing and carboxyl-terminal amidation of the nonapeptide. Like the oxytocin-family precursors in tetrapods and coelacanth, the elephant shark oxytocin does not contain a copeptin (Figs 3 and 5). Thus, these precursors are different from the oxytocin-family precursor in teleost fishes which contains a copeptin moiety. However, the teleost fish oxytocin-family precursor does not contain an arginine residue between the neurophysin and the copeptin (Fig 4 and 5), and therefore, the copeptin may not be cleaved into a separate moiety.

### No evidence for gene conversion between elephant shark vasotocin and oxytocin genes

The second exons of human and bovine *vasotocin *and *oxytocin *genes that encode the central region of the neurophysin exhibit an unusually high level of sequence identity (Table 2). In the case of bovine genes, in addition to 197 bp of the exonic sequence, 135 bp of the preceding intron is also totally conserved. This observation had led to the hypothesis that the two genes have experienced a recent gene conversion event [15]. We have previously reported that the second exons of the coelacanth *vasotocin *and *mesotocin *genes also show a high degree of sequence identity at the amino acid (97%) and nucleotide (98%) levels [8]. However, we have shown that these exons do not have a high GC3 content which is a signature of a gene conversion event, and therefore proposed that the high sequence conservation is the result of purifying selection acting on the nucleotide sequences rather than due to a gene conversion event [8].

**Table 2:** GC content of the third codon positions (GC3) in the three coding exons of elephant, coelacanth, bovine and human neurohypophysial hormone genes.

		Exon 1	Exon 2	Exon 3
Human	vasopressin gene	92.5%	95.5%	98.2%
	oxytocin gene	90.0%	94.0%	88.9%

Bovine	vasopressin gene	87.5%	94.0%	93.3%
	oxytocin gene	90.0%	95.5%	89.5%

Coelacanth	vasotocin gene	42.5%	41.2%	48.1%
	mesotocin gene	40.0%	41.2%	56.3%

Elephant shark	vasotocin gene	60.0%	72.1%	57.1%
	oxytocin gene	70.0%	69.1%	68.4%

Lamprey	vasotocin gene	76.2%	76.2%	73.5%

The second exons of the elephant shark *vasotocin *and *oxytocin *genes also exhibit a high level of sequence identity at the amino acid (95.1%) and nucleotide (97.1%) levels. Moreover, the high level of sequence identity extends into 14 bp of the 5' intron and 6 bp of the 3' intron (Fig 3). To determine whether these exons have experienced a gene conversion, we estimated the GC3 content of all the three exons of the two genes. The GC3 content of the second exons of the two genes is in fact lower (69 and 72%) than that of their homologs in mammals (94 to 95.5%), and is comparable to that of their respective first exons (60 and 70%) and third exons (57 and 68%) (Table 2). Furthermore, the GC3 content of the second exons is comparable to that of the three exons of the single *vasotocin *gene in the Japanese lamprey (73.5% to 76.2%; Table 2). Together, these data suggest that there is no evidence for gene conversion between the second exons of the elephant shark *vasotocin *and *oxytocin *genes and that the GC3 content of the second exons of these genes merely reflects the GC3 content of the ancestral *vasotocin *gene in the lamprey. It is therefore likely that the high identity of the second exons of the elephant shark *vasotocin *and *oxytocin *genes is also due to purifying selection acting at the nucleotide level similar to that on the second exons of the coelacanth *vasotocin *and *mesotocin *genes. This implies that the second exons encode a functional element besides coding for amino acids. This conserved element may be involved in transcript stability, microRNA binding [16], enhancing splicing [17] or regulation of transcription that requires the sequence to be highly conserved at the nucleotide level.

### Expression patterns of elephant shark *vasotocin *and *oxytocin *genes

**Figure 6:**
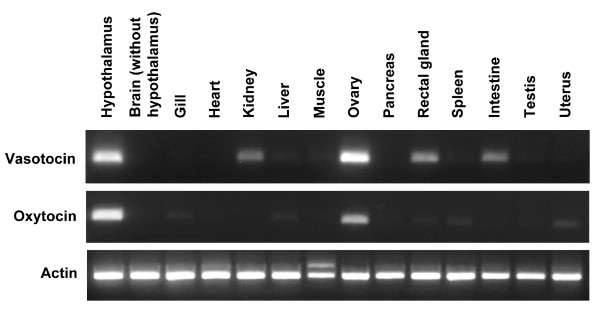
**Expression patterns of elephant shark neurohypophysial hormone genes**. Expression patterns of the elephant shark *vasotocin *and *oxytocin *genes as determined by semi-quantitative RT-PCR. Expression of *actin *gene was analyzed as a control for the quality of RNA and cDNA.

We determined the expression patterns of the elephant shark *vasotocin *and *oxytocin *genes by a semi-quantitative reverse transcription PCR. Single strand cDNA was prepared from about one microgram of total RNA each from hypothalamus, brain (excluding hypothalamus), gills, heart, kidney, liver, muscle, ovary, pancreas, rectal gland, spleen, intestine, testis and uterus; and PCR was carried out using the same volume of cDNA preparation from different tissues. The results therefore indicate the relative levels of expression of each gene between various tissues. Interestingly, besides the hypothalamus, both *vasotocin *and *oxytocin *genes were found to express in some peripheral tissues indicating that they play a paracrine role in these tissues. Vasotocin was found to express at relatively high levels in the ovary and at low levels in the kidney, rectal gland and intestine and oxytocin was found to express at moderate levels in the ovary (Fig 6). To the best of our knowledge, no such peripheral expression of neurohypophysial hormone genes has been reported in teleost fishes or in non-mammalian tetrapods. However, it is well known that these genes express in peripheral tissues in some mammals. For example, *oxytocin *gene is expressed in the uterine epithelium, placenta, amnion and intrauterine tissues in rats; in the amnion, chorion, decidua intrauterine tissues and cumulus cells surrounding the oocytes in humans; and in the corpus luteum and testis in cows [18]. The *vasopressin *gene is expressed in the aorta [19,20], pancreas [19] and testis in rats [21]. Consequently, it has been proposed that these hormones play a paracrine role in mammals. The osmoregulatory system in marine cartilaginous fishes involves the kidney, gills, rectal gland and gastrointestinal tract. The kidney excretes water, salt and nitrogenous wastes such as urea and trimethylamine oxide; the gills eliminate nitrogen in the form of ammonia; and the gut epithelium reabsorbs water, salt and nutrients. The rectal gland is a highly specialized salt-secreting organ unique to marine cartilaginous fishes [22]. The expression of *vasotocin *gene in the elephant shark kidney, rectal gland and intestine suggests that vasotocin plays a paracrine role in the osmoregulatory functions of these tissues. In mammals, oxytocin is known to induce smooth muscle contraction in the ovary and uterus and thereby play a role in ovulation and parturition [23]. In addition, oxytocin synthesized in the human cumulus cells has been suggested to play a role in fertilization and early embryonic development [18]. In elephant shark, fertilization and partial embryonic development occurs internally ('ovoviviparous'). Mating normally occurs before the spawning season and females store sperm is a special pouch until the eggs mature. Eggs mature in batches and the stored sperm is used for fertilization as and when the eggs mature. During spring, females migrate into shallow bays and inlets and lay eggs in shallow soft sediment habitats. Each female lays two fertilized eggs at a time every 7 to 8 days over a period of two to three months [24]. The elephant sharks for this study were collected in shallow waters during the peak spawning season. The expression of both *oxytocin *and *vasotocin *genes in the elephant shark ovary during this period suggests a role for these hormones in ovulation (i.e., release of mature oocytes from the follicles) and/or in 'parturition' (release of fertilized eggs) in this ovoviviparous vertebrate. *In vivo *and *in vitro *studies on the effects of vasotocin and oxytocin should provide evidence to support this hypothesis.

### Neurohypophysial hormone gene locus in the Japanese lamprey

The living jawless vertebrates are represented by the lampreys and hagfishes. So far only a *vasotocin *gene has been cloned in these vertebrates [1,25]. However, it is not known if they also contain an oxytocin-family gene. To determine this we sequenced the neurohypophysial hormone gene locus in the Japanese lamprey. We screened a Japanese lamprey BAC library using a probe for the *vasotocin *gene and identified one positive BAC clone (#191L19). This BAC was sequenced completely. It contains a 90-kb insert (GenBank accession number FJ195978) that encodes three complete genes: *vasotocin*, *Gnrh2 *and *Ptpra*. This locus contains only 4.7% repetitive sequences, with LINEs and SINEs contributing to 0.5% and 0.4%, respectively (Fig 2B). Thus the amount of repetitive sequences in the lamprey locus is considerably lower than that in the homologous loci in the elephant shark (42% repeats) and coelacanth (17% repeats) [8]. The lamprey vasotocin gene comprises three exons and two introns like the vasopressin- and oxytocin-family genes in jawed vertebrates. The relative order and orientation of the three genes in the lamprey locus are identical to that of their homologs in elephant shark, coelacanth, *Xenopus*, chicken, and opossum. However, the lamprey locus lacks an oxytocin-family gene in the intergenic region between the vasopressin-family gene and the *Gnrh2 *gene that is present in these vertebrates (Fig 1B). Although there is a possibility that a oxytocin-family may be present elsewhere in the lamprey genome, previous screenings of cDNA libraries from the Japanese lamprey and hagfish [1,25] were successful in identifying only a *vasotocin *gene in these vertebrates. We therefore conclude that jawless vertebrates do not contain an oxytocin-family gene present in jawed vertebrates.

### Neurohypophysial hormone gene locus in amphioxus

**Figure 7:**
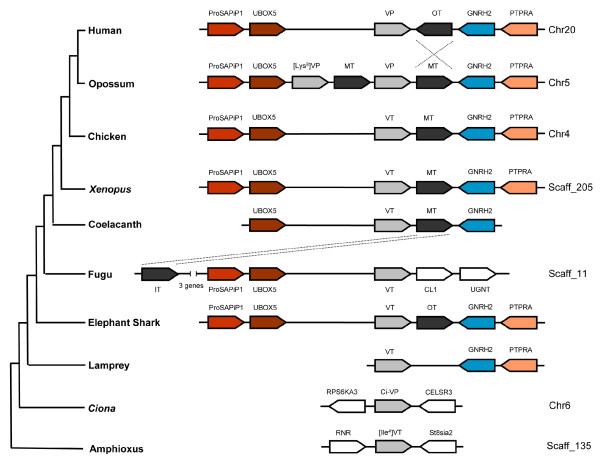
**Schematic diagram of neurohypophysial hormone gene loci in chordates**. Arrows represent genes and indicate the orientation of transcription. ProSAPiP1, *ProSAP-interacting protein 1 *gene; UBOX5, *U-box domain containing 5*; [Lys^8^]VP, *lysipressin *gene; VT, *vasotocin *gene; MT, *mesotocin *gene; OT, *oxytocin *gene; IT, *isotocin *gene, GNRH2, *gonadotropin-releasing hormone 2*; PTPRA, *protein tyrosine phosphatase receptor type A *gene; CL1, chemokine CL1 gene; UGNT, UDP glucuronic acid/N-acetylglucosamine dual transporter gene; RNR, *ribonucleotide reductase M2 polypeptide*; St8sia2, *alpha-2,8-sialyltransferase*; RPS6KA3, *ribosomal protein S6 kinase 90kDa polypeptide3*; Ci-VP, *Ciona-vasopressin*; and CELSR3, *cadherin EGF LAG seven-pass G-type receptor3*.

The living chordates are classified into three major lineages, the cephalochordates (e.g., amphioxus), urochordates (tunicates) and vertebrates. The cephalochordates are the most basal group of chordates and the urochordates are the sister group of vertebrates [26]. Recently a gene encoding a vasopressin-related peptide has been cloned in the urochordate, *Ciona intestinalis*. This gene comprises three exons and two introns like the vasopressin- and oxytocin-family genes in vertebrates. However, unlike the nonapeptides encoded by the vertebrate genes, the *Ciona *gene codes for a 13-amino acid peptide hormone (designated as Ci-vasopressin; Table 3[27-38]) that lacks a carboxyl-terminal amidation signal (Gly-Lys-Arg) [39]. Interestingly, in another urochordate, *Styela plicata*, a gene encoding an oxytocin-like peptide has been cloned. This gene also comprises three exons and two introns but codes for a 14-amino acid peptide hormone (Table 3). It however contains a typical carboxyl-terminal amidation signal. This prohormone is designated as Styela oxytocin-related peptide (SOP) [40]. To date no neurohypophysial hormone gene has been cloned in cephalochordates. Recently, a whole-genome sequence of a cephalochordate, the amphioxus has been completed [26]. We searched for neurohypophysial hormone genes in the amphioxus genome assembly by TBLASTN algorithm using the elephant shark and lamprey neurohypophysial hormone precursor protein sequences as queries. Only one gene with a high similarity to vasopressin-family peptide was identified. We annotated the coding exons based on homology to the elephant shark and lamprey neurohypophysial hormone genes, and refined the exon-intron boundaries by manual inspection. The amphioxus gene comprises three exons and two introns, with the positions and phases of introns being identical to that of the vasopressin- and oxytocin-family genes in vertebrates. The 167-amino acid prohormone encoded by this gene consists of a signal peptide, a nonapeptide hormone, neurophysin and copeptin similar to the vasotocin and vasopressin prohormones in vertebrates (Fig 4). The nonapeptide is linked to the neurophysin molecule by a typical tripeptide sequence (Gly-Lys-Arg) that is known to act as a signal for proteolytic processing and C-terminal amidation of the hormone. The nonapeptide hormone, however, contains Ile at the 4^th ^position unlike the vertebrate vasotocin and vasopressin peptides which contain a Gln at this position (Fig 4). We therefore designate the amphioxus peptide as [Ile^4^]vasotocin. The amphioxus copeptin does not contain an N-glycosylation site similar to the copeptin in teleost fishes and lamprey. It also lacks a Leucine-rich core segment that is present in the copeptin of all vertebrates (Fig 4). The presence of a typical nonapeptide neurohypophysial hormone in amphioxus indicates that urochordates (*Ciona *and *Styela*) are the only exception among metazoans that do not contain a typical nonapeptide hormone (see Table 3). The amphioxus [Ile^4^]*vasotocin *gene is flanked by *RNR *and *ST8sia2 *genes, which are unrelated to the genes flanking the neurohypophysial hormone genes in vertebrates (Fig. 7). The *Ciona *neuropeptide gene, *Ci-vasopressin*, is flanked by genes (*RPS6KA3 *and *CELSR3*) that are unrelated to the genes flanking the neurohypophysial hormone genes in vertebrates as well as in amphioxus (Fig. 7). Thus, the synteny of genes in amphioxus is not conserved in *Ciona*, and the synteny of genes in vertebrates is conserved neither in amphioxus nor in *Ciona*.

**Table 3:** Neurohypophysial hormones characterized in invertebrates

**Name**	**Peptide**	**Organism**	**Reference**
Annetocin	Cys-Phe-Val-Arg-Asn-Cys-Pro-Thr-Gly (NH_2_)	Earthworm (*E. foetida*)	[27]

Lys-conopressin	Cys-Phe-Ile-Arg-Asn-Cys-Pro-Lys-Gly (NH_2_)	Pond snail (*Lymnaea stagnalis*)	[28,29]
		
		Geography cone (*Conus geographus*)	[30]
		
		Sea hare (*Aplysia kurodai*)	[31]
		
		Leech (*Erpbdella octoculata*)	[32]
		
		Imperial cone (*Conus imperialis*)	[33]

Arg-conopressin	Cys-Ile-Ile-Arg-Asn-Cys-Pro-Arg-Gly (NH_2_)	Striped cone (*Conus striatus*)	[30]

Cephalotocin	Cys-Phe-Ile-Arg-Asn-Cys-Pro-Ile-Gly (NH_2_)	Octopus (*Octopus vulgaris*)	[42]

Octopressin	Cys-Phe-Trp-Thr-Ser-Cys-Pro-Ile-Gly (NH_2_)	Octopus (*Octopus vulgaris*)	[41]

Inotocin	Cys-Leu-Ile-Thr-Asn-Cys-Pro-Ile-Gly (NH_2_)	Locust (*Locusta migratoria*)	[34]
		
		Red flour beetle *(Tribolium castaneum)*	[35,36]
		
		Parasitic wasp *(Nasonia vitripennis)*	[36]

Crustacean oxytocin/vasopressin-like peptide	Cys-Phe-Ile-Thr-Asn-Cys-Pro-Pro-Gly (NH_2_)	Water flea *(Daphnia pulex)*	[36]

*Ciona *VP/oxytocin-related peptide (Ci-VP)	Cys-Phe-Phe-Arg-Asp-Cys-Ser-Asn-Met-Arp-Trp-Tyr-Arg	Sea squirt (*Ciona intestinalis*)	[39]

*Styela *oxytocin-related peptide (SOP)	Cys-Tyr-Ile-Ser-Asp-Cys-Pro-Asn-Ser-Arg-Phe-Trp-Ser-Thr (NH_2_)	Potato sea squirt (*Styela plicata*)	[40]

[Ile^4^]vasotocin	Cys-Tyr-Ile-Ile-Asn-Cys-Pro-Arg-Gly (NH_2_)	Amphioxus *(Branchiostoma floridae)*	This study

### Origin and evolutionary history of vertebrate neurohypophysial hormone genes

Neurohypophysial hormones are an ancient family of hormones with representatives found in diverse taxa among invertebrates and vertebrates. However, invertebrates contain either a vasopressin-family peptide or an oxytocin-family peptide (Table 3) but seldom both peptides. Although some invertebrates like octopus contain two peptides, cephalotoxin and octopressin, both are oxytocin-like peptides [41,42]. In contrast to invertebrates, all jawed vertebrates contain at least one member each of vasopressin- and oxytocin-family peptides. In jawless vertebrates, which occupy an intermediary position between invertebrates and jawed vertebrates, only a vasotocin gene has been cloned so far. In this study, we sequenced the neurohypophysial gene loci in a cartilaginous fish, the elephant shark, and a jawless vertebrate, the Japanese lamprey. We also characterized this locus in the genome of amphioxus, a cephalochordate. Our study shows that while both amphioxus and lamprey contain a single neurohypophysial hormone gene that encodes a basic vasopressin-family peptide, elephant shark contains both a vasopressin-family gene and a oxytocin-family gene that are closely linked tail-to-head (Fig 7). These data suggest that the two families of peptides arose in a common ancestor of jawed vertebrates through a tandem duplication of the ancestral *vasotocin *gene. The coding sequence of the duplicate gene has subsequently diverged to code for a neutral peptide thereby giving rise to the oxytocin-family of neurohypophysial hormone. The different patterns of expression of *vasotocin *and *oxytocin *genes in elephant shark also imply that, in addition to mutations in the protein coding sequence, the regulatory region of the duplicated gene(s) has also undergone changes to confer a different pattern of expression to the two daughter genes. Investigation of the expression pattern of *vasotocin *gene in lamprey should shed light on the ancestral expression pattern, and how it has diverged in the daughter genes. Unfortunately, due to the unavailability of RNA from various tissues of the Japanese lamprey, we could not determine the expression pattern of the *vasotocin *gene in the lamprey.

The tail-to-head orientation of the elephant shark *vasotocin *and *oxytocin *genes indicates that this was the ancestral state of the two genes soon after the duplication of the *vasotocin *gene. The close linkage and organization of these genes have been conserved in coelacanth, *Xenopus*, chicken and opossum genomes while the *oxytocin *gene has undergone a local inversion in human and rodent genomes (Fig 7). In contrast to these vertebrates, the neurohypophysial hormone gene locus in pufferfishes has undergone extensive rearrangements. The *isotocin *gene in fugu has been translocated to a position upstream of *vasotocin *gene and the two genes are separated by five unrelated genes. In addition, the two genes located downstream of the *vasotocin *gene in fugu (*CL1 *and *UGNT*) are unrelated to the genes present at a similar position (*Gnrh2 *and *Ptpra*) in other vertebrates (Fig. 7). A search for the neurohypophysial hormone genes in the genome assemblies of other teleost fishes such as the stickleback, medaka and zebrafish on the UCSC Genome Browser [30] revealed that while the arrangements of *isotocin *and *vasotocin *genes in stickleback (ChrXIII) and medaka (Chr9) are similar to that in fugu, the two genes are located on separate chromosomes (Chr5 and Chr8 respectively) in zebrafish. These data indicate that the neurohypophysial hormone gene locus has undergone extensive rearrangements in teleost fishes, in contrast to its well conserved synteny in other vertebrates including the jawless lamprey. These findings are consistent with previous observations that teleost fish genomes have experienced a higher rate of chromosomal rearrangements compared to other vertebrates (reviewed in [43]). The rearrangements in teleost fishes might be related to the "fish-specific" whole genome duplication that occurred in the teleost ancestor [44,45]. The duplicated gene loci generated by this whole genome duplication might have facilitated rearrangements between paralogous chromosomal segments through homologous recombination.

We would like to add that our efforts to build a phylogenetic tree of invertebrate and vertebrate neurohypophysial hormone genes resulted in an anomalous gene tree in which the vasopressin- and oxytocin-family genes in fugu, elephant shark, coelacanth and mammals clustered with each other suggesting that the two genes originated independently in each lineage (Additional file 1). This is highly unlikely and is an artifact of the phylogenetic analysis due to the highly conserved sequences of these two paralogous genes. For such paralogous genes, the conservation of synteny in different taxa is a much better indicator of their orthologous relationships.

## Conclusion

We have characterized the neurohypophysial hormone gene locus in elephant shark (a cartilaginous fish), Japanese lamprey (a jawless vertebrate) and amphioxus (a cephalochordate) and showed that amphioxus and lamprey each contains a single gene belonging to the vasopressin-family while elephant shark contains a vasopressin-family and an oxytocin-family gene that are closely linked in a tail-to-head orientation. These results indicate that vasopressin- and oxytocin-family peptides evolved in a common ancestor of jawed vertebrates through tandem duplication of the ancestral *vasotocin *gene. The *vasotocin *and *oxytocin *genes in elephant shark exhibit distinct expression patterns. Thus, this is a classical example for the origin of a novel gene with a distinct function and expression pattern through duplication of an ancestral gene. The synteny and order of genes in the neurohypophysial hormone gene locus are conserved in lamprey, elephant shark, coelacanth and tetrapods, but disrupted in teleost fishes presumably due to the rearrangements facilitated by a whole genome duplication event in the teleost fish ancestor.

## Methods

### Isolation of elephant shark BAC clone

The 1.4× elephant shark genome sequence assembly  was searched using TBLASTN algorithm and human vasopressin and oxytocin precursor protein sequences as queries. The search identified three scaffolds (AAVX01010109.1, AAVX01174980.1 and AAVX01018755.1) that contained sequences with high similarity to the human *vasopressin *and *oxytocin *genes. Searching these scaffold sequences against non-redundant protein sequence database at NCBI using BLASTX showed that they contained gene fragments for vasotocin and oxytocin hormone precursors. A pair of primers (VTF 5'-CTG TTC CAG TGT TTG CCG TGT-3' and VTR 5'-TAC CAT CAT CAC AGC AGA TTC C-3') complementary to the second exon of the putative *vasotocin *gene fragment in scaffold AAVX01174980.1 was used to amplify a 217-bp fragment by PCR from the elephant shark genomic DNA. The PCR cycling conditions consisted of an initial denaturation step at 95°C for 2 min, followed by 35 cycles of 95°C for 30 sec, 60°C for 1 min and 72°C for 30 sec, with a final elongation step at 72°C for 5 min. The PCR product was gel-purified using GeneClean (Qbiogene, Irvine, USA), labeled with [α-32P] dCTP using Random Primed DNA labeling kit (Roche Diagnostics, Mannheim, Germany) and used as a probe to screen an elephant shark BAC library (IMCB_Eshark BAC library cloned in pCCBAC-EcoRI). Six positive BAC clones (#65F8, #113K9, #157M2, #191N1, #208M19, #209O22) were identified. Two of these clones (#191N1 and #208M19) were selected for sequencing. Comparing the sequences of the three elephant shark scaffolds that we identified using human vasopressin precursor protein sequence to the sequence of these BACs showed that they all belong to this locus.

### Isolation of lamprey BAC clone

Using the following primers (LPF 5'-ATC TGC TGC GGG GAG GCC ATG GG-3' and FPR 5'-CAG GCC GGG AGC TCC RCA YTT-3') complementary to the coding sequence of the lamprey *vasotocin *gene (BAA06669), a 140-bp fragment of the *vasotocin *gene was amplified from the Japanese lamprey genomic DNA. An 'overgo' probe was prepared for this sequence by using a pair of oligonucleotides with an 8-bp overlap at their 3' ends (LPF 5'-ATC TGC TGC GGG GAG GCC ATG GG-3' and LPR2 5'-CAC CCA GGC GAC AGC CCA TGG C-3'). The overlapping oligonucleotides were mixed in equal proportion (10 pmol/μl of each) and annealed by incubating at 80°C for 5 min, followed by incubation at 37°C for 10 min and then transferred to ice. The annealed oligonucleotides were extended and labeled with [α-^32^P]dATP and [α-^32^P]dCTP by primer extension with Klenow at room temperature for 30 min to produce a 36-bp dual-labeled overgo probe. The unincorporated nucleotides were removed by passage through a Sephadex-G50 column (Amersham, Piscataway, NJ, USA). The overgo probe was then used to screen a Japanese lamprey BAC library (IMCB_Japanese lamprey BAC library, cloned in pCCBAC-EcoRI; average insert size, ~100 kb; unpublished) and only one positive BAC clone (#161L19) was identified, presumably due to an uneven representation of the genome.

### Sequencing and assembly of elephant shark and lamprey BAC clones

Two of the elephant shark BAC clones (#191N1 and #208M19) and the single lamprey BAC clone (#161L19), were sequenced completely using the shotgun sequencing strategy. Briefly, BAC DNA was fragmented by hydrodynamic shearing (Hydroshear, GeneMachines, San Carlos, CA) and then end-repaired by Klenow treatment. Fragments in the size range of 2–3 kb were gel purified and subcloned into the *Eco*RV site of pBluescript SK vector. High quality sequences were acquired by sequencing both ends of plasmid inserts using standard BigDye Terminator v3.1 chemistry on an ABI 3730xl DNA analyzer. Raw shotgun DNA sequence reads were quality-trimmed and assembled with Phred/Phrap/Consed software package . Gaps were filled by 'primer-walking' using BAC DNA as a template or by sequencing bridge clones or by sequencing PCR products.

### Sequence analysis

Protein-coding sequences were predicted based on homology to known proteins in the non-redundant protein database at the National Centre for Biotechnology Information using BLASTX and TBLASTN algorithms [32]. Exon-intron boundaries were refined by manual inspection. The genomic sequences of the neurohypophysial hormone gene locus for human (March 2006 assembly), *Xenopus tropicalis *(assembly version 4.1), chicken (assembly version 2.1), gray short-tailed opossum (Jan 2006 assembly), fugu (assembly version 4.0), *Tetraodon nigroviridis *(February 2004 assembly) and amphioxus (JGI ver.1.0) were obtained from the UCSC Genome Browser while that of coelacanth was retrieved from GenBank (accession number EU284132). Multiple sequence alignments of protein sequences were carried out with ClustalX Version 1.83 [46]. Repetitive sequences were identified using the RepeatMasker (version open-3.1.6) [31] and GIRI Repbase [47].

### RT-PCR

Total RNA was isolated from the elephant shark tissues using TRIzol reagent (Invitrogen, USA). About one microgram of total RNA from each tissue was reverse transcribed using SuperScript™ First-Strand Synthesis System for RT-PCR (Invitrogen) according to the manufacturer's instructions. The single strand cDNA was resuspended in 50 μl of water and one microliter was used for PCR. PCR primers were designed to amplify across exons 2 and 3 of the neurohypophysial hormone genes. Care was taken to avoid regions of high conservation. Elephant shark actin was amplified as an internal control for the quality of RNA and cDNA.

The following primers were used in PCR: vasotocin (sense, 5'-CAC CGG GAA TCT GCT GTG ATG-3'; antisense, 5'-GAT ACC GCC TGG TAC TTC TTTG-3'), oxytocin (sense, 5'-GCT CAG AGC GTG GCA AGT-3'; antisense, 5'-GGT CCA CGG CAC AGC TTT-3'), and actin (sense, 5'-GGG TAT TGT CAC CAA CTG GGAC-3'; antisense, 5'-TCT ACC CGT GTC ACA CCC AC-3'). PCR amplification cycles include an initial denaturation at 95°C for 2 min, followed by 35 cycles of 95°C for 30 sec, 55°C for 30 sec and 72°C for 1 min, followed by an extension step at 72°C for 5 min.

## Authors' contributions

BV and SB conceived and designed the project. PG carried out sequencing, annotation and analysis of the sequences reported. BT screened the BAC library, carried out cDNA synthesis and RT-PCR, and helped in sequencing BAC clones. PG and BV wrote the manuscript. All authors read and approved the final manuscript.

## Supplementary Material

Additional file 1 **Neighbor-Joining tree of protein sequences of invertebrate and vertebrate vasopressin- and oxytocin-family of hormones**. Numbers at the nodes are bootstrap values of 1000 replicates. The paralogous vasopressin- and oxytocin-family genes in each taxon are erroneously clustered with each other (marked with a circle). eshark, elephant shark; VP, vasopressin; OT, oxytocin; MT, mesotocin; VT, vasotocin; IT, isotocin; SOP, *Styela *oxytocin-related peptide. Click here for file
