# Supplementary figures and images for: Characterization of the neurohypophysial hormone gene loci in elephant shark and the Japanese lamprey: origin of the vertebrate neurohypophysial hormone genes
Source: BMC Evol Biol. 2009 Feb 26;9:47. doi: 10.1186/1471-2148-9-47 (PMC2656470; doi:10.1186/1471-2148-9-47)

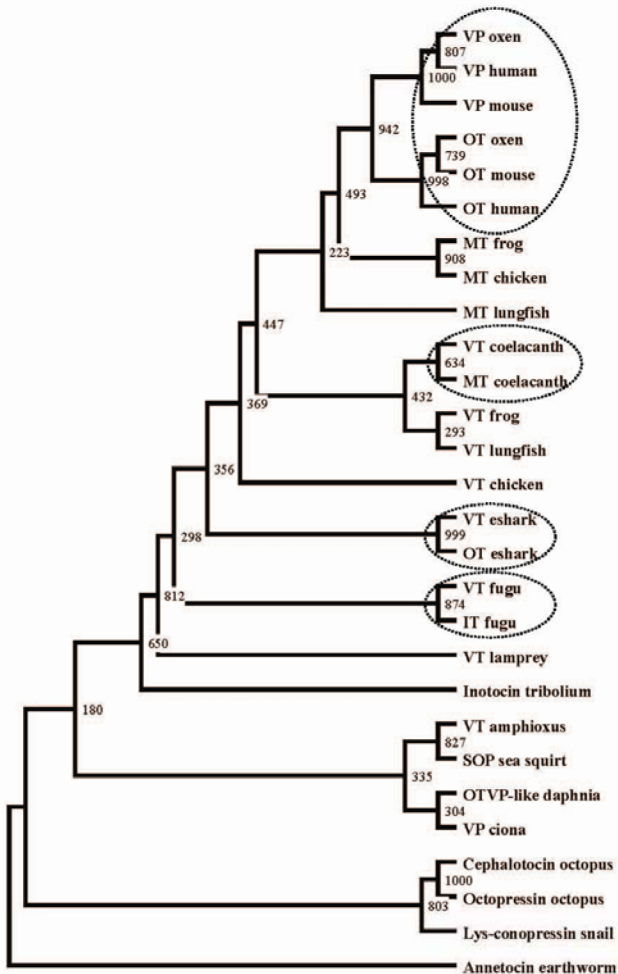

Supplement: Additional file 1 — Neighbor-Joining tree of protein sequences of invertebrate and vertebrate vasopressin- and oxytocin-family of hormones. Numbers at the nodes are bootstrap values of 1000 replicates. The paralogous vasopressin- and oxytocin-family genes in each taxon are erroneously clustered with each other (marked with a circle). eshark, elephant shark; VP, vasopressin; OT, oxytocin; MT, mesotocin; VT, vasotocin; IT, isotocin; SOP, Styela oxytocin-related peptide. [file 1471-2148-9-47-S1.pdf]
